# Natural Melanin-Based Nanoparticles With Combined Chemo/Photothermal/Photodynamic Effect Induce Immunogenic Cell Death (ICD) on Tumor

**DOI:** 10.3389/fbioe.2021.635858

**Published:** 2021-02-19

**Authors:** Ding Liu, Huilin Huang, Bingxia Zhao, Weihong Guo

**Affiliations:** ^1^Department of Organ Transplantation, Zhujiang Hospital, Southern Medical University, Guangzhou, China; ^2^Department of General Surgery, Nanfang Hospital, Southern Medical University, Guangzhou, China; ^3^Guangzhou Key Laboratory of Tumor Immunology Research, School of Basic Medical Sciences, Cancer Research Institute, Southern Medical University, Guangzhou, China

**Keywords:** immunogenic cell death, melanin, natural product, photothermal, photodynamic, ROS, DOX

## Abstract

Melanin, as a natural product, has been used as an extraordinary ingredient for nanomedicine due to its great biocompatibility and light responsive property. In this study, polydopamine (PDA), an analog of melanin, was extracted from dopamine and encapsulated with doxorubicin (DOX). The as-prepared nanoparticles (NPs) with good stability, great biosafety and high near infrared (NIR) responsive property ameliorated the cell uptake of DOX in OS-RC-2/ADR cells, exhibited synergistic chemo/photothermal (PTT)/photodynamic (PDT) effects, induced the release of damage associated molecular patterns (DAMPs), and finally, led to immunogenic cell death (ICD). In general, it was suggested that PDA-DOX NPs with NIR irradiation could serve as a promising agent for tumor therapy.

## Introduction

Cancer is a challenging health issues for human beings, with 14 million new cases and over eight million deaths worldwide every year (Bray et al., 2018). As the investigation of tumor characteristics continues, a number of potent cancer fighting strategies have been successfully adopted in clinical practice. Among these combating methods, chemotherapy remains the most preferred and remarkable treatment. For example, doxorubicin (DOX) can not only restrain the proliferation and metastasis of tumor cells, but can also simultaneously lead to immunogenic cell death (ICD) (Casares et al., 2005). ICD, as a promising treatment, aims at enhancing anti-tumor immunity, controlling and damaging cancer cells, and sensitizing therapy by immune system activation. Antineoplastic chemotherapeutic agents can alter the tumor microenvironment that has been infiltrated by various immune cells; whose characteristics usually determine the therapeutic outcome. So, it is prospectively used to facilitate antitumor chemo-immunotherapy (Casares et al., 2005; Obeid et al., 2007; Lu et al., 2018). However, their clinical use is greatly compromised by their adverse systemic effects arising from poor specificity on cancerous tissue. Furthermore, the therapeutic effect could be dramatically reduced owing to drug resistance and an adverse tumor microenvironment. Clinicians often choose to increase the dosage of drugs or change the therapy scheme when topical drug concentration in the cancer region is reduced. Nevertheless, this method could also lead to systemic toxicity, such as liver damage, bone marrow suppression, neuritis, and other unknown adverse events. As a result, there is an urgent need to explore the deeper mechanisms of drug resistance and to find other ways of improving therapeutic efficiency.

Numerous DOX-based nanomedicines have been synthesized to enhance the absorption of DOX at the cancer region, by taking advantage of the enhanced permeability and retention (EPR) effect. However, the therapeutic performance of these nanomedicines remains unsatisfactory. Whether chemotherapeutic resistance is inherent or acquired (Dean et al., 2005; Iyer et al., 2013; Pérez-Herrero and Fernández-Medarde, 2015; Liang et al., 2016) is one of the greatest challenges of effective therapy. Consequently, nanomedicines incorporated into additional therapeutic modalities have the potential to yield better clinical benefits. In recent years, phototherapy based on near infrared (NIR) coupled with chemotherapy, has been developed as a desirable treatment strategy due to its precise tumor localization, highly efficient ablating capability, and better biocompatibility (Zhu et al., 2018). Phototherapy can accelerate the release of drugs to a deeper part of the tumor, by increased vascular permeability and reverse drug resistance (Li et al., 2015, Li F. et al., 2017; Huang et al., 2019). On the other hand, the integrity and permeability of the cancer cell membrane would be damaged by phototherapy, which can also stimulate the release of tumor-related antigens and can activate the immune response, namely ICD (Sweeney et al., 2018; Li et al., 2019; Shang et al., 2020; Wang et al., 2020). Detecting a proper photosensitive agent for cementing between chemotherapy and phototherapy to achieve ICD and therapy sensitization is therefore of great urgency.

Melanin and its analogs are distributed in many creatures, and widely utilized as ubiquitous biomaterials, owing to their optical absorption property, photoconversion, and affinity. They are wonderful nanocarriers applied to the field of biological imaging, phototherapy, antioxidant therapy, and drug delivery systems, etc. (Solano, 2017; Huang et al., 2018). Moreover, it is worth noting that melanin-like nanoparticles (NPs) can modulate an immune response, as it has been elaborated that NPs from cuttlefish ink mediated the repolarization of M2 TAMs to M1 (Ye et al., 2017; Deng et al., 2019; Rong et al., 2019). As a result, melanin and melanin-like nanoparticles are suitable nanoplatform for drug loading, phototherapy, and immune activation.

**SCHEME 1:**
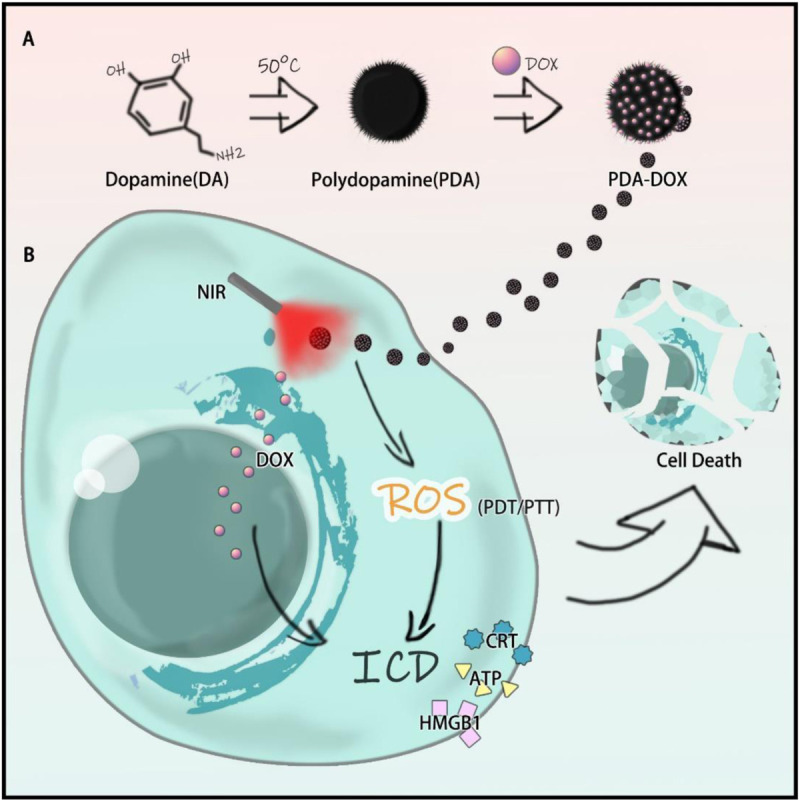
The mechanism of PDA-DOX with NIR inducing ICD in OS-RC-2/ADR cells.

Polydopamine (PDA) was synthesized, as one kind of melanin-like NPs, while the characteristics and the biocompatibility of PDA and DOX loading PDA (PDA-DOX) NPs were also detected. Synergistic antitumor efficiency of PDA-DOX NPs with NIR was investigated on cancer cells. More importantly, it was found that PDA-DOX NPs could induce the release of damage associated molecular patterns (DAMPs) leading to ICD (as shown in Scheme 1).

## Materials and Methods

### Materials and Characterization

Dopamine hydrochloride (98%), Sodium hydrate (99%), and Poly-(ethylene glycol) (NH_2_-mPEG-NH_2_ MW 2000, 98%) were obtained from Guangzhou Tanshui Co., Ltd (Guangzhou, China). Deionized (DI) water (18.2 MΩ cm), obtained from a water purification system (Synergy, Millipore, MA), was used in all preparation processes. Transmission electron microscope (TEM) images were taken by a JEOL JEM-2100F TEM. Zeta potential and hydrodynamic diameter measurement was performed by Zetasizer Nano ZS (Malvern). Shimadzu UV-2600 UV–vis spectrophotometer was used to acquire UV–vis absorption spectra. Nicolet/Nexus 670 Fourier transform infrared (FTIR) Analyzer (Thermo Nicolet, United States) was used to obtain FTIR spectra.

### Synthesis of Different Sizes of PDA NPs

300 mg of dopamine hydrochloride (1.95 mmol) (Aladdin) were dissolved in 216 mL of deionized water. 1,700 μL of 1 mol/L NaOH solution was added to a dopamine hydrochloride solution at 50°C under vigorous stirring. When the solution’s color turned to pale yellow, NaOH was added into the solution, and gradually the color changed to dark brown. After stirring for 6 h, the solution was further centrifuged with a centrifugal-filter (Amicon centrifugal filter device, MWCO = 10 kDa) and washed with deionized water; this was repeated three times.

### Surface Modification of PDA NPs With NH2-PEG_5000_-NH_2_ (PEG-Melanin-Like NPs)

1 mol/L NaOH solution was added to 5 mL of melanin-like aqueous solution (5 mg/mL of water) to adjust the pH of the solution to 9. This mixed solution was added dropwise into a 25 mg NH_2_-PEG_2000_-NH_2_ aqueous solution with pH = 9. After vigorous stirring for 8 h, PEG-modified PDA NPs was retrieved by centrifugation with a centrifugal-filter (Amicon centrifugal filter device, MWCO = 10 kDa), followed by washing with deionized water several times to remove the unreacted NH_2_-PEG_2000_-NH_2_. Finally, the aqueous solvent was removed by freeze-drying, and a PEG-PDA-like powder was obtained.

### Photothermal (PTT)/Photodynamic (PDT) Effects of PDA-DOX NPs

PDA-DOX NPs were treated by 808 nm wavelength laser irradiation (0.7 W/cm^2^, 5 min), as a thermal probe was used to detect the temperature changes at different time-points, while an equivalent amount of PBS with the same laser irradiation was chosen as the negative control. The images of temperature changes were recorded by an infrared imaging device (ThermaCAMSC3000, Flirsystem Incorporation, United States) at 0.5 min internals for a total of 5 min. To further validate PDT potentials, the yield of ROS produced by PDA-DOX NPs under NIR (0.7 W/cm^2^) was quantitatively analyzed by DPBF. The absorption value of DPBF and PDA-DOX NPs mixed solution at 410 nm was detected every 1 min.

### Drug Loading Efficiency of PDA-DOX NPs

Doxorubicin and PDA NPs was mixed at ratios of 1:0.125, 1:0.25, 1:0.5, and stirred in the dark at room temperature for 24 h. The formed NPs were centrifuged with a centrifugal-filter (Amicon centrifugal filter device, MWCO = 30 kDa) and washed with deionized water to remove unloaded DOX. Unloaded DOX was collected and analyzed using a UV–vis–NIR spectrophotometer. The loading content [weight of loaded DOX/(weight of loaded DOX + weight of NPs) × 100%] and loading efficiency (weight of loaded DOX/weight of added DOX × 100%) of DOX on the NPs was calculated.

### pH-Responsive Drug Release of PDA-DOX NPs

10 mg PDA NPs loaded with DOX were resuspended in 10 mL deionized water. The samples were transferred into a dialysis membrane bag with a MWCO of 3,500, which was immersed in 30 mL buffer at 5.5, 6.5 and 7.4, respectively. At predetermined time points, 3 mL of release medium was taken out and 3 mL fresh buffer was added. The content of released DOX was measured by a PerkinElmer UV750 spectrophotometer (PE Co., United States) at a wavelength of 480 nm.

### Cell Culture and Preparation

Human renal proximal tubule epithelial cell (HK-2) cells and OS-RC-2/ADR cells (American Type Culture Collection) were bought, cultured, and maintained by Dulbecco’s modified Eagle’s medium (DMEM; Gibco, Langley, OK, United States) supplemented with 10% fetal bovine serum (FBS; Gibco) and antibiotics (100 U/mL) at 37°C in 5% CO_2_. For the preparation of experiments, cells were seeded into 6-well plates or 96-well plates, respectively, and incubated with appropriate DMEM added with 10% FBS. The prepared cells were exposed to the DMEM with 10% FBS (*the blank control*), DOX (*the DOX group*), PDA (*the PDA group*), and PDA-DOX (*the PDA-DOX group*) at different concentrations. After incubation with these nanomaterials, half of the treated cells were irradiated with an 808 nm laser (*NIR group*) (0.7 W/cm^2^, 5 min), and all continued to incubate for further experiments.

### Confocal Laser Scanning Microscope Imaging

OS-RC-2/ADR cells were seeded in confocal laser scanning microscope (CLSM) dishes, cultured for 24 h in DMEM supplemented with 10% FBS. Cells were then incubated with PDA-DOX NPs for 1–4 h. The cells were then washed, fixed by 4% paraformaldehyde, and stained with DAPI. Finally, the dishes were imaged by CLSM (Olympus, Japan).

### Flow Cytometry Assay

OS-RC-2/ADR cells were seeded in 6-well plates, cultured for 24 h in DMEM supplemented with 10% FBS. Cells were then incubated with PDA-DOX NPs for 1–4 h, harvested, suspended, and analyzed by flow cytometry. Furthermore, in our study, the fluorescence channel of DOX and PE is similar so that the PE-positive cells were considered to have internalized DOX, and flow cytometry assays were also used to assess the amount of Reactive Oxygen Species (ROS).

### Cell Counting Kit-8 Assay

HK-2 cells were cultured for 24 h in DMEM supplemented with 10% FBS before the incubation with PDA-DOX, at the varying concentrations for 24 h. Then a Cell Counting Kit-8 (CCK-8) detection kit was applied to prove the biosafety of PDA-DOX NPs following the protocol. The biosafety was assumed by the cell viability ratio of exposed groups to the blank control (cells exposed to the DMEM with 10% FBS).

To detect the anti-tumor effect of PDA-DOX NPs, OS-RC-2/ADR cells were seeded into 96-plates, cultured for 24 h in DMEM supplemented with 10% FBS. After that, cells were exposed to different treatments, and cell viabilities were analyzed using CCK-8 assay.

### Live and Death Assay

The antitumor effect of PDA-DOX with/without NIR was assessed by Live and Death Staining Kit (KeyGen, Nanjing, China). OS-RC-2/ADR cells were plated and cultured overnight. Then, cells were incubated with different concentrations of DOX, PDA, and PDA-DOX, respectively. Following the above treatment, cells were subsequently treated with the absence or presence of NIR irradiation (0.7 W/cm^2^, 5 min). Next, as per the manufacturer’s instruction of Live and Death Staining Kit, treated cells were labeled as green (live) or red (dead), and finally monitored by a confocal laser scanning microscope (CLSM, Olympus).

### 5-Ethinyl-2′ DNA Nucleoside Uracil (EdU) Assay

The antitumor effect of PDA-DOX with/without NIR was assessed by 5-ethinyl-2′ DNA nucleoside uracil (EdU) assay (KeyGen, Nanjing, China). OS-RC-2/ADR cells were plated and cultured overnight. Cells were then incubated with the same concentration of DOX, PDA, and PDA-DOX, respectively. Following the above treatment, cells were subsequently treated with or without NIR irradiation (0.7 W/cm^2^, 5 min). Next, as per the manufacturer’s instruction of EdU assay, treated cells were labeled, fixed, stained, and finally monitored by a confocal laser scanning microscope (CLSM, Olympus). The three random zones were captured to calculate the number of EdU-positive cells.

### ROS Assay

OS-RC-2/ADR cells were seeded into a 6-well plate and cultured for 24 h (37°C, 5% CO2). After that, cells were incubated with the same concentrations of DOX, PDA, and PDA-DOX for 48 h respectively, with or without the presence of NIR irradiation (0.7 W/cm^2^, 5 min). Finally, cells were incubated with ROS Assay Kit (KeyGen, Nanjing, China).

### *In vitro* Detection of ICD Biomarkers

The treated OS-RC-2/ADR cells were collected and fixed with 4% paraformaldehyde for 10 min. After being blocked for 1 h at room temperature by 5% BSA, the cells were further incubated with rabbit anti-Rabbit chaperone calreticulin (CRT) or large amounts of high-mobility group box 1 (HMGB1) primary antibodies, respectively, at 4°C overnight. The cells were subsequently incubated with PE or FITC-labeled goat anti-rabbit secondary antibodies away from light for an additional 1 h. Finally, the nuclei were stained with DAPI and the CRT and HMGB1 expression levels were observed under CLSM (Olympus, Japan). Additionally, the HMGB1 ELISA kit was used to detect the release of HMGB1 in the supernatant, while the Luminescent ATP Detection Assay was applied to evaluate the release of adenosine triphosphate (ATP) in the supernatant.

### Statistical Analysis

Mean ± standard deviation (SD) was used to value the data. All experiments were repeated at least three times, unless indicated otherwise. The unpaired Student’s *t*-test or the analysis of variance (ANOVA) followed by Scheffe’s *post hoc* test was applied to value the data. A *P* < 0.05 was considered to be a significant difference.

## Results and Discussion

### Synthesis and Characterizations of PDA-DOX

**FIGURE 1:**
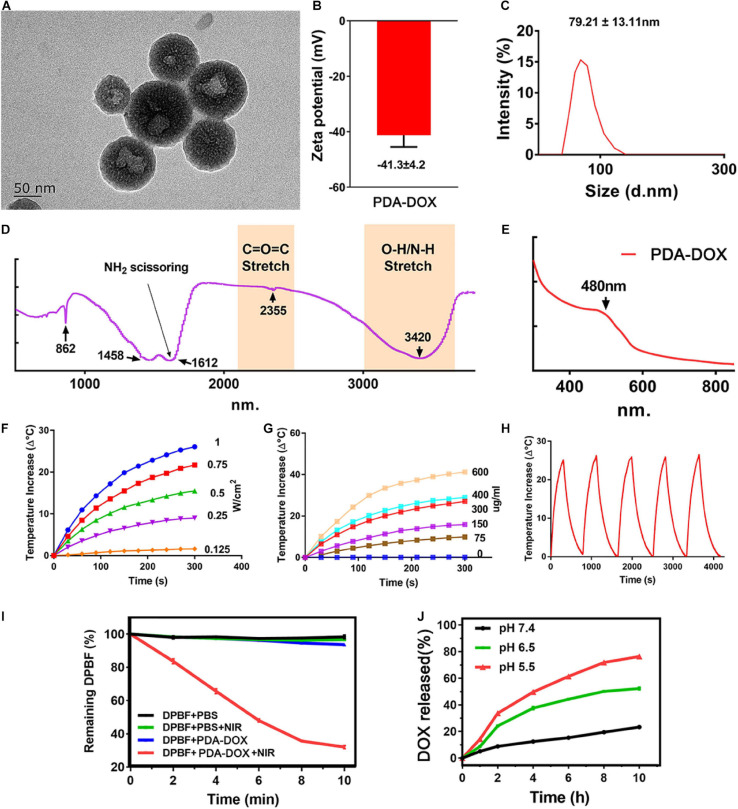
**(A)** TEM image, **(B)** Zeta potentials, **(C)** DLS, **(D)** FT-IR, and **(E)** UV results of PDA-DOX NPs. **(F)** The temperature increase curve induced under different powers of NIR irradiation. **(G)** The temperature increase curve induced by different concentrations of PDA-DOX NPs and PBS. **(H)** PTT stability of PDA-DOX NPs under NIR irradiation (0.7 W/cm^2^). **(I)** The production of ^1^O_2_ by PDA-DOX NPs with or without NIR irradiation (0.7 W/cm^2^). **(J)** The pH-responsive release curve of PDA-DOX.

Melanin, as a natural product, has been used for cancer phototherapy due to its great biocompatibility and NIR light responsive properties. In this study, a cancer phototherapy nanoplatform was obtained based on PDA, by oxidation–polymerization of dopamine monomers in alkaline environments. The different sizes were synthesized from ∼80 to 370 nm (Supplementary Figure 1) by simply adjusting the pH value, as the size of PDA would decrease with the increase of the pH value (Hawley et al., 1967; Ju et al., 2011). The antitumor drug, DOX was loaded into PDA NPs (named as PDA-DOX) for the purpose of synergetic chemotherapy, as DOX can be loaded onto PDA by means of π-π conjugation and coordination (Li W. Q. et al., 2017). The DOX loading capacity (DLC) increased with the amount of feeding DOX, and a DLC as high as 67% was obtained when the feeding DOX vs. PDA (w/w) was 1:0.125 (Supplementary Figure 2A). However, the DOX loading efficiency (DLE) gradually decreased as the feeding DOX/PDA mass ratios increased (Supplementary Figure 2B). Therefore, considering the economical utilization efficiency, we chose the feeding ratio of 1:0.25 (DOX *vs.* PDA) for the following experiments. To further improve the water solubility, PEG was applied to modify the surface of PDA NPs. The successful synthesis of PDA-DOX NPs was characterized by TEM, zeta potential, UV–vis spectra analysis, and FTIR analysis. PDA-DOX NPs exhibited an average size distribution of 79.21 ± 27.11 nm with a negative charged surface zeta potential of −41.3 ± 4.2 eV, as demonstrated by the TEM, zeta potential and DLS results (Figures 1A–C). The broad absorption bands shown in the ATR-FTIR analysis of PDA-DOX NPs between 3,690 and 3,000 cm^–1^ are characteristics of the O–H or N–H stretching vibration modes. These broad absorption bands were resulted by carboxylic acid, phenolic, and aromatic amino functions present in the indolic and pyrrolic systems (Ozlu et al., 2019). At 1,612 cm^–1^, NH_2_ scissoring could be seen, representing the successful modification of PEG on PDA-DOX NPs (Figure 1D).

Apart from that, several studies have reported that melanin and its analogs could respond well to NIR light (650–900 nm), in most conditions transferring light into heat (Baldea et al., 2018; Deng et al., 2019; Rong et al., 2019; Xiong et al., 2019). The NIR-responsive properties of PDA-DOX NPs were then measured by irradiating with a NIR laser (808 nm). It could be seen that the temperature increases of the PDA-DOX NPs solution showed both concentration and laser power density dependent properties, indicating their good photothermal effect (Figures 1F,G). Moreover, the temperature increase remained almost the same even after five laser on/off cycles, demonstrating their good photothermal stability (Figure 1H). Furthermore, in our previous works (Hou et al., 2018; Chen et al., 2019; Guo et al., 2020) it has been found that some photothermal agents also possess the ability to generate ROS under NIR irradiation and could be used as photosensitizers for photodynamic therapy. Herein, ROS generation ability of our as-prepared NPs was also investigated using a ROS probe DPBF. As seen from Figure 1I, the absorption of DPBF decreased most in the PDA-DOX-treated group than in the other groups, suggesting that more ^1^O_2_ could be produced when the NPs were irradiated by NIR laser. The above results indicate that the PDA-DOX NPs could be applied as a promising PTT and photodynamic therapy (PDT) agent.

The encapsulation of DOX was characterized by UV–vis spectra. It could be seen that a distinctive absorbance peak appeared at approximately 480 nm of the PDA-DOX NP compared with PDA NPs only, indicating the successful encapsulation of DOX (Figure 1E). The loading efficiency of DOX was calculated as 30%. The drug release property of PDA-DOX NPs was also evaluated under different pH values (pH 7.4, pH 6.5, and pH 5.5). It could be seen that only a small amount of DOX would be released at a neutral pH value, which could decrease the side effects to the normal tissues. The drug was released more rapidly at lower pH values and exhibited an obvious pH-dependent drug release performance (Figure 1J). Considering the acid tumor microenvironments, the pH-sensitive dox release makes the PDA-DOX NPs a promising DOX delivery system.

### Cellular Uptake and Biosafety of PDA-DOX *in vitro*

**FIGURE 2:**
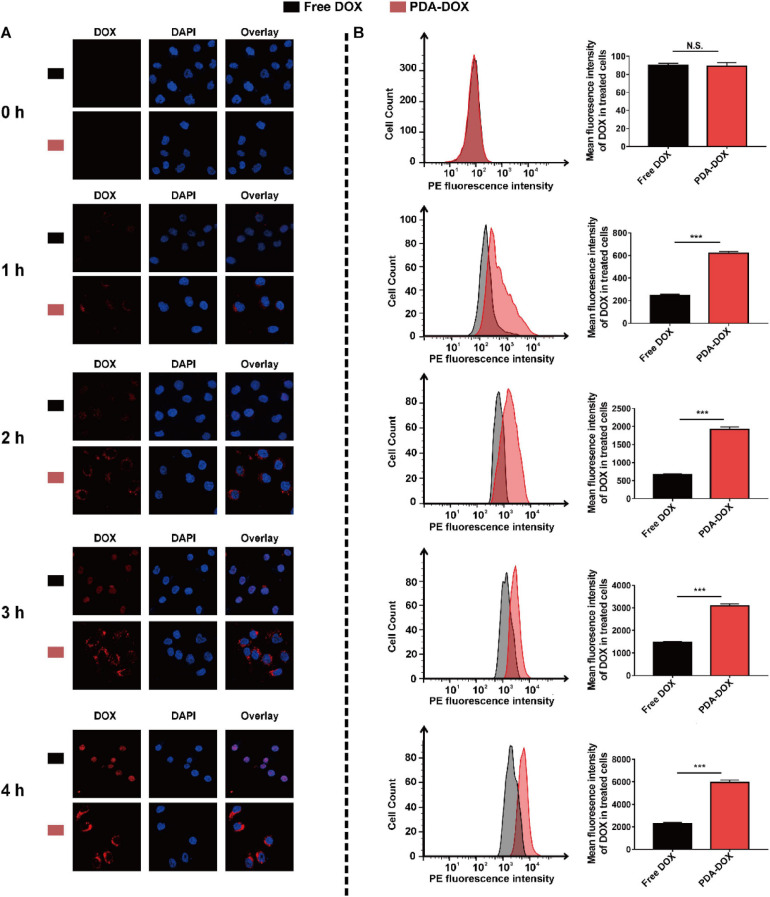
Cell uptake of PDA-DOX analyzed by **(A)** CLSM and **(B)** flow cytometry. *** indicates *P* < 0.001. N.S. means no significance.

As a natural product, it has already been proven that melanin and its analogs can easily enter cancer cells, making them a widely used drug delivery platform (Ju et al., 2013; Jiang et al., 2017). It is therefore expected that cellular uptake of DOX in cancer cells could increase greatly using PDA nanocarriers. To confirm the synergistic uptake of PDA-DOX, OS-RC-2/ADR cells were exposed to DOX and PDA-DOX separately from 0 to 4 h and analyzed with CLSM and flow cytometry. CLSM showed that red fluorescence intensity from DOX increased as time passed, both in DOX and PDA-DOX groups. It could be observed that the fluorescence intensities in PDA-DOX group were stronger than those in the free DOX group at different time points (Figure 2A), indicating that PDA indeed helps to gain a more desired cell uptake ratio of DOX. Correspondingly, the accumulation of DOX in OS-RC-2/ADR cells treated with free DOX and PDA-DOX was also analyzed by flow cytometry. The result revealed that cellular uptake of DOX at 4 h was threefold that of free DOX (Figure 2B), indicating that the as-prepared PDA-DOX NPs could significantly enhance the cellular uptake of DOX.

In addition, a prerequisite of nanoparticles for therapy is that they are less toxic to normal cells (Huang et al., 2018). Hence, the CCK-8 assay was used to evaluate the biosafety of PDA-DOX to normal HK-2 cells, while over 85% cell viability was observed, after HK-2 cells were exposed to PDA-DOX for 24 h with the increasing PDA concentrations up to 200 μg/mL (Supplementary Figure 3). Overall, these results revealed that PDA-DOX NPs have great biocompatibility and could be used to delivery DOX into renal cancer cells for treatment.

### PDA-DOX NPs With Combined Chemo/PTT/PDT Effect on Killing Cancer Cells

**FIGURE 3:**
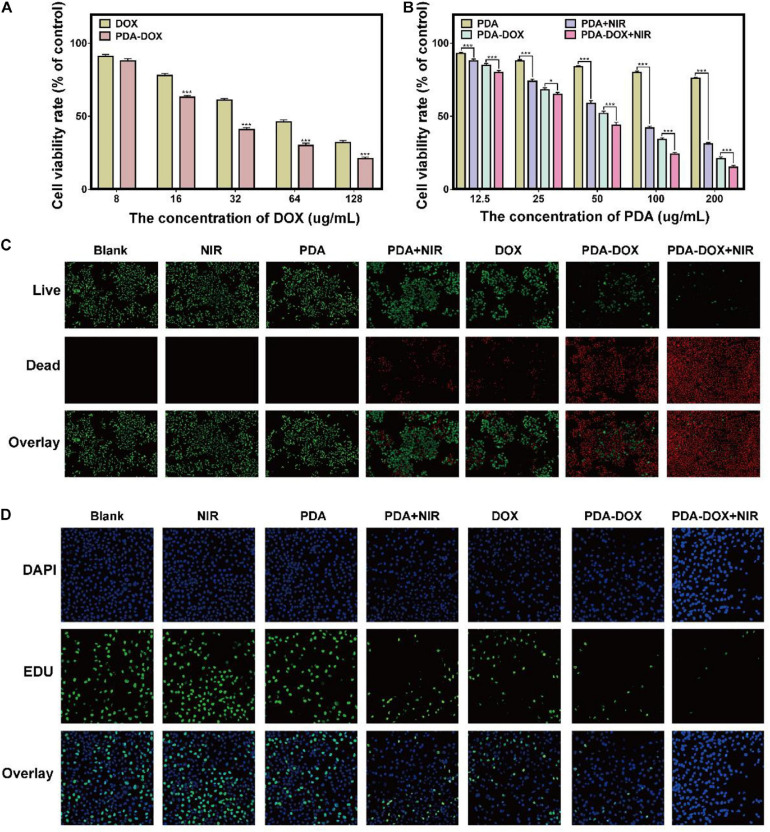
The antitumor effect of PDA-DOX *in vitro*. **(A)** Viabilities of OS-RC-2/ADR cells after incubation with DOX and PDA-DOX at different concentrations of DOX. **(B)** Viabilities of OS-RC-2/ADR cells after incubation with PDA and PDA-DOX, with or without NIR at different concentrations of PDA, while the DMEM with 10% FBS was used as the negative control (blank) group. **(C)** Live/Dead Cell Double Staining images of OS-RC-2/ADR cells after the incubation with PDA, DOX, and PDA-DOX, at presence or absence of NIR. **(D)** EdU images of OS-RC-2/ADR cells after the incubation with PDA, DOX, and PDA-DOX, upon NIR or not. Data are shown as the mean ± SD, *n* = 3. *** indicates *P* < 0.001. *indicates *P* < 0.05.

As mentioned above, PDA gives rise to high cellular uptake of DOX and exhibit a pH-sensitive drug release property, which could theoretically increase the chemo-therapeutic effect of DOX. Therefore, better antitumor effects could be achieved when PDA NPs were used as the nanocarrier with the same DOX concentration (Figure 3A). As proven above, the intrinsic NIR-responsive properties of the PDA NPs make them a potential PTT/PDT agent. For example, it has been reported that PDA, functioned with arginine-glycine-aspartic-cysteine acid (RGDC) peptide and loaded with DOX, could be released and induces chemo-photothermal effect availably (Li Y. et al., 2017). It could therefore be hypothesized that PDA-DOX with NIR irradiation can cause not only a chemical lesion but also thermal injury and oxidative stress. To further validate the synergistic chemo/PTT/PDT effect of our as-prepared NPs, OS-RC-2/ADR cells were incubated with the PDA, DOX, and PDA-DOX at different concentrations, respectively, with or without the presence of NIR (0.7 W/cm^2^, 5min). After being cultured for 24 h, treated OS-RC-2/ADR cells were analyzed by CCK-8, EdU and Live/Dead Cell Double Staining Kit. As illustrated in Figure 3B, cell viabilities decreased with the increasing concentrations of DOX and PDA-DOX, while PDA-DOX with NIR-irradiation could achieve the best therapeutic effect compared with PDA-and-NIR-irradiation-treated cells, PDA-DOX-treated cells, and the control group. Moreover, EdU assay revealed that PDA-DOX-and-NIR-irradiation-treated, PDA-and-NIR-irradiation-treated, and PDA-DOX-treated cells were shown to be less EdU-positive in comparison with the control and DOX-treated groups (Figure 3C). A similar tendency was also observed in Live/Dead Cell Double Staining Kit results (Figure 3D). These results suggested that PDA-DOX could facilitate the chemo-effectiveness of DOX, and in combination with NIR would stimulate better efficiency than mono chemotherapy or phototherapy, implying a PDA-DOX induced synergetic chemo/PTT/PDT effect against OS-RC-2/ADR cells.

### Immunogenic Cell Death Induced by Synergetic Chemo/PTT/PDT Effect of PDA-DOX *in vitro*

**FIGURE 4:**
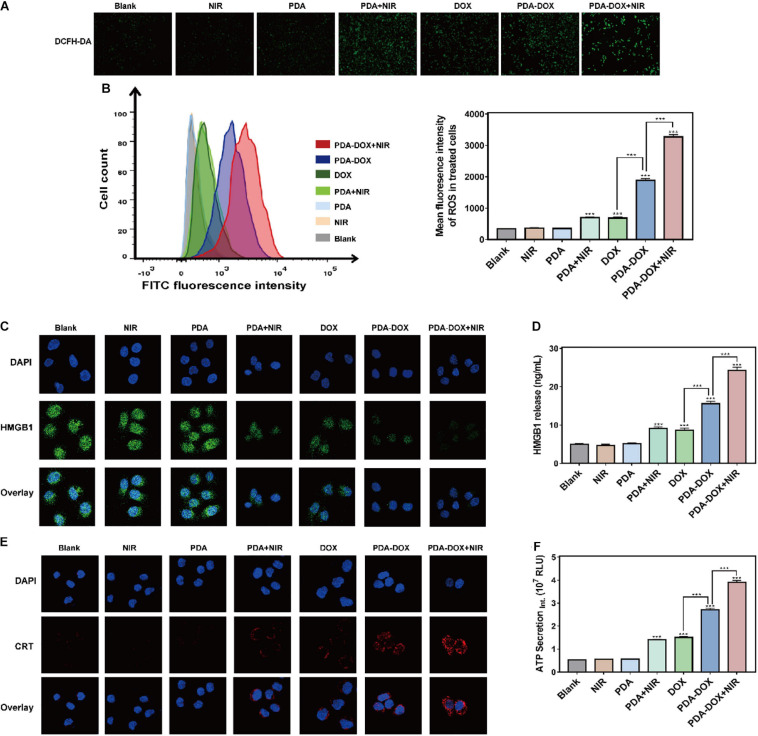
ICD induced by synergetic chemo/PTT/PDT effect of PDA-DOX *in vitro*. **(A,B)** The generation of ROS in OS-RC-2/ADR cells after the incubation with PDA, DOX, and PDA-DOX with or without NIR. **(C)** HMGB1 images of OS-RC-2/ADR cells after the incubation with PDA, DOX, and PDA-DOX with or without NIR. **(D)** The HMGB1 release of OS-RC-2/ADR cells after incubation with PDA, DOX, and PDA-DOX with or without NIR. **(E)** CRT images of OS-RC-2/ADR cells after the incubation with PDA, DOX, and PDA-DOX with or without NIR. **(F)** The ATP secretion of OS-RC-2/ADR cells after incubation with PDA, DOX, and PDA-DOX with or without NIR. Data are shown as the mean ± SD, *n* = 3. *** indicates *P* < 0.001.

Immunogenic Cell Death is a kind of regulated cell death and is able to distinguish from other cell death so that it can activate the immune system against tumor cells (Ahmed and Tait, 2020; Fumet et al., 2020). It has been reported that DOX has the immunogenicity to recruit immune cells according to the ICD, but the efficiency of mono DOX is not strong enough (Casares et al., 2005). Recent studies found that phototherapy based on melanin and its derivatives might cause ICD, consecutively reinforcing immune response and more tumor cell death (Yan et al., 2019; Li et al., 2020). During this process, ICD plays an important initial role and DAMPs are provoked into release to promote the immunostimulatory effect. Three typical DAMPs have been recognized in virtually all ICD inducers, including CRT, ATP, and HMGB1 (Kepp et al., 2014). Moreover, it has been confirmed that the release of DAMPs, especially CRT’s translocating into membrane, results from endoplasmic reticulum (ER) stress related to the generation of extra ROS (Garg et al., 2011; Galluzzi et al., 2012; Gomes-da-Silva et al., 2018; Deng, 2020). Considering this, we assumed that increasing ROS might be key in leading to ICD effects of PDA-DOX under NIR irradiation. Interestingly, it conformed with our hypothesis that stronger ROS means that fluorescence intensity could be observed in the PDA-DOX-and-NIR-irradiation-treated group rather than in other groups (Figures 4A,B). The extra generation of ROS might be rooted in the combined chemo/PTT/PDT effect of PDA-DOX due to its high light absorption.

Consequently, we attempted to clarify whether the combination of PDA-DOX and NIR can stimulate ROS production to boost the release of the above DAMPs and ICD of DOX or PDA with NIR alone. CLSM imaging then revealed that DOX, PDA-DOX, PDA plus DOX, and PDA-DOX plus NIR, could induce translocation of CRT into the cell membrane, while the PDA-DOX plus NIR group led to more obvious CRT translocation (Figure 4E). In addition, we observed the downregulated expression level of HMGB1 inside the cell (Figure 4C), and more HMGB1 release and ATP secretion in the supernatant after treatment with PDA-DOX plus NIR (Figure 4D,F), indicating that ICD could be elicited by PDA-DOX plus NIR. Taken together, these results suggest that PDA-DOX with NIR irradiation do not only facilitate the release of DOX to attract tumor cell death, but also results in more ICD effects in contrast to DOX or PDA with NIR. It is the same as previous research, where DOX combined with NIR irradiation resulting in ICD. It is presumed that high-efficient ICD is attributed not only to a high concentration of local DOX but also phototherapy (Phung et al., 2019; Wen et al., 2019; Xia et al., 2019; Zhang et al., 2019). In summary, this shows that the synergetic chemo/PTT/PDT effect of PDA-DOX and NIR irradiation could lead to the ICD *via* the generation of ROS, providing prospects for the combination with tumor immunotherapy.

## Conclusion

In this work, we developed PDA-DOX NPs, the natural product used for chemotherapy drug loading, to improve the effect of DOX. It not only inherited great stability and sound biocompatibility of the natural product, but also exhibited strong photothermal conversion ability and a simultaneous ROS generation effect, which is appropriate for phototherapy. Considering this, PDA-DOX plus proper NIR irradiation *in vitro* showed good synergistic chemo/PTT/PDT effects against OS-RC-2/ADR cells, compared to DOX or PDA with NIR alone. Furthermore, boosting ROS is a key process to mediating the release of DAMPs, involving CRT, HMGB1, and ATP. In conclusion, PDA-DOX might be a dramatic drug for renal carcinoma chemotherapy and phototherapy, and is promising for tumor immunotherapy.

## Data Availability Statement

The original contributions presented in the study are included in the article/Supplementary Material, further inquiries can be directed to the corresponding author/s.

## Author Contributions

WG and BZ designed the study, revised the figures and tables, and contributed to drafting the manuscript. DL and HH collated the data, carried out data analyses, and produced the initial draft of the manuscript. All authors read and approved the final manuscript.

## Conflict of Interest

The authors declare that the research was conducted in the absence of any commercial or financial relationships that could be construed as a potential conflict of interest.
