# Supplementary material for: Natural Melanin-Based Nanoparticles With Combined Chemo/Photothermal/Photodynamic Effect Induce Immunogenic Cell Death (ICD) on Tumor
Source: Front Bioeng Biotechnol. 2021 Feb 19;9:635858. doi: 10.3389/fbioe.2021.635858 (PMC7935529; doi:10.3389/fbioe.2021.635858)
Supplement: Supplementary file 1 [file Table_1.DOCX]

Supplementary materials

**Method and materials**

**The construction of DOX resistance cells: OS-RC-2/ADR cells**

OS-RC-2 cells were exposed to doxorubicin (DOX) at an initial concentration of 0.025 mg/L. Subsequently, the survival cells which were tolerant to DOX, were selected for further cultured in a double concentration of doxorubicin. This procedure was repeatedly applied to the cells, until the cells could finally be cultured well in the presence of 1.0 mg/L doxorubicin. When the cells were totally tolerance to doxorubicin, the ADR cells were successfully established. The ADR cells were continuously exposed to certain doxorubicin concentrations for resistance maintenance.

**
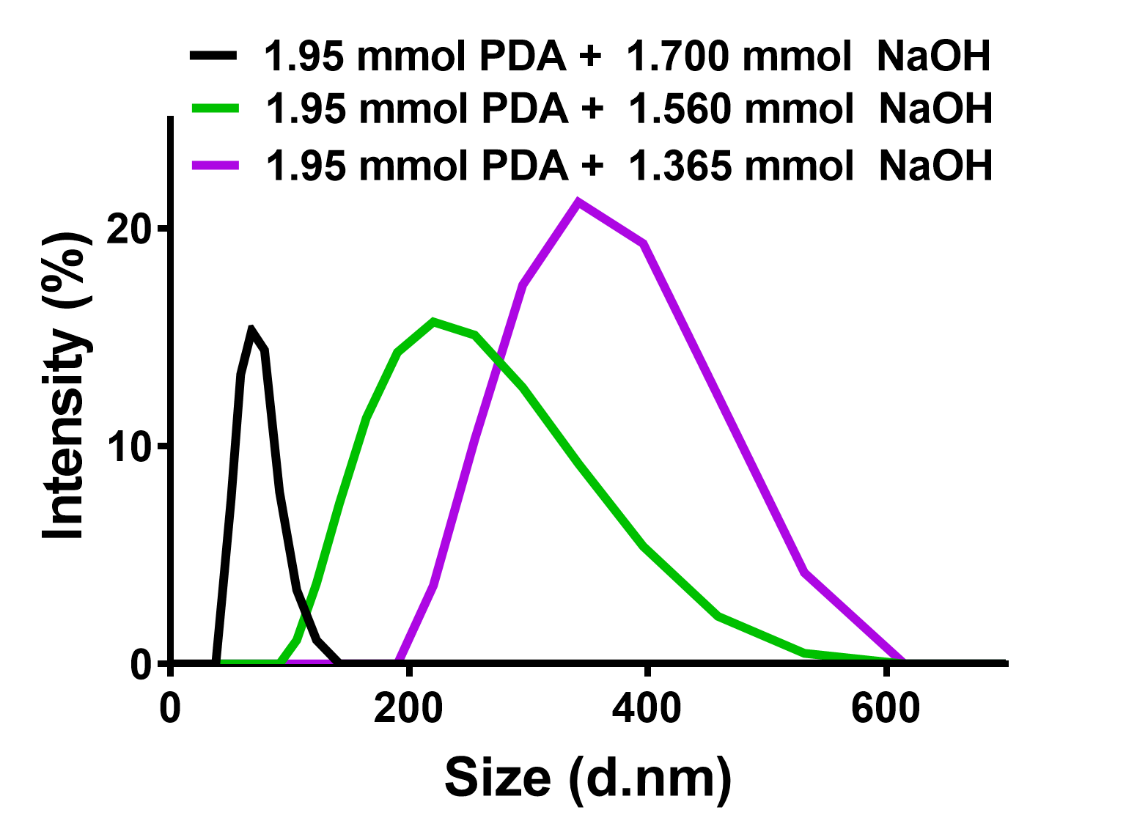
Results**

Figure S1. Different sizes of PDA NPs at varying amount of NaOH

**
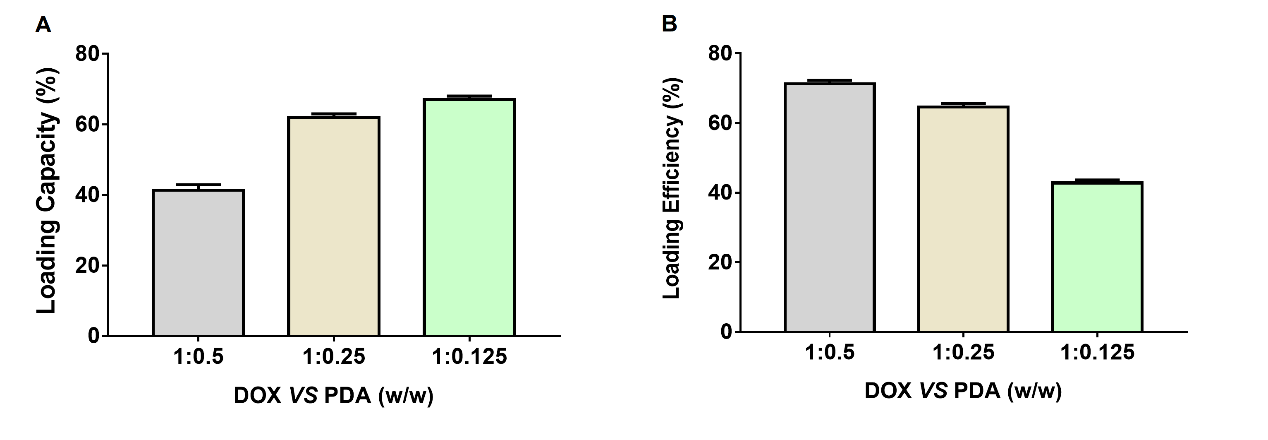
**Figure S2(A)&(B). Loading capacity and loading efficiency of PDA-DOX at ratio of 1:0.5, 1:0.25 and 1:0.125, respectively.


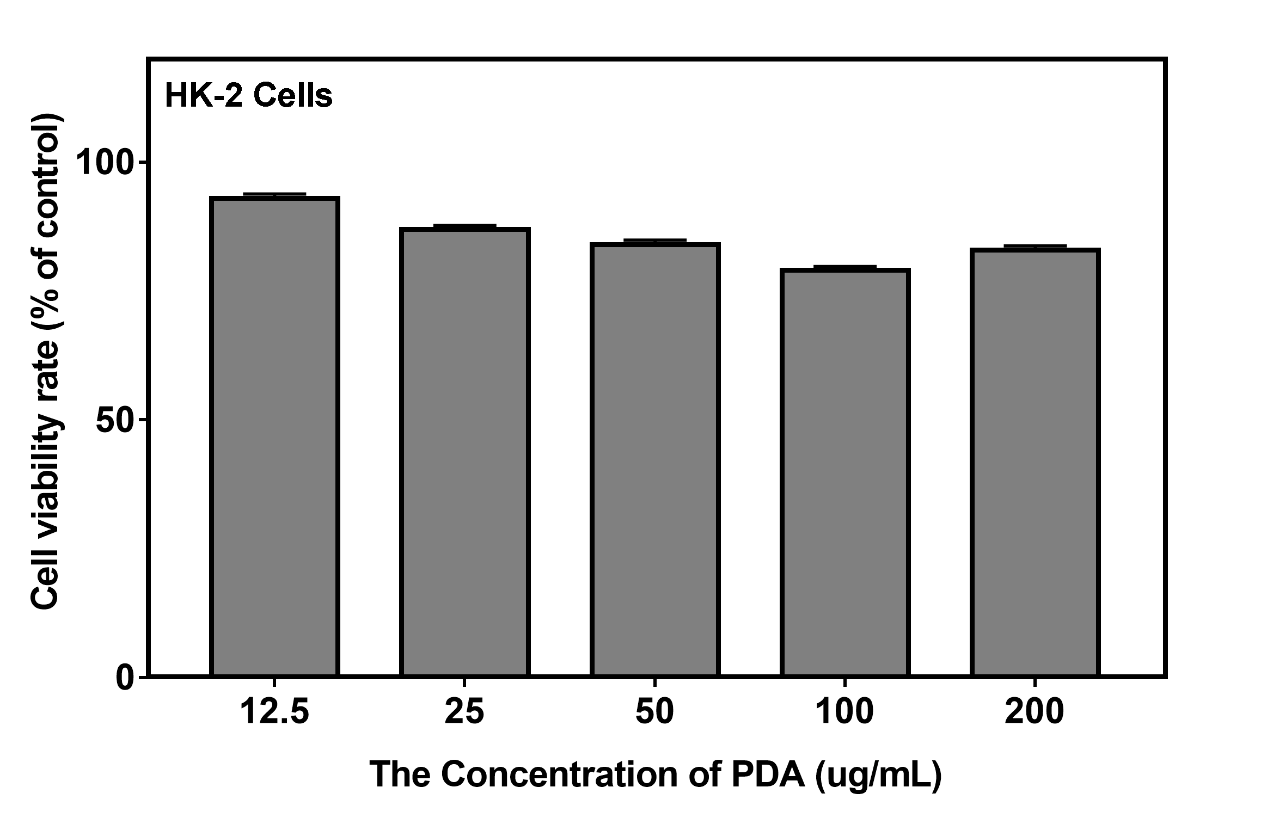
Figure S3. Viabilities of HK-2 cells after incubation with PDA-DOX at different concentrations of PDA
